# *Cleistocalyx nervosum* var. *paniala* Berry Promotes Antioxidant Response and Suppresses Glutamate-Induced Cell Death via SIRT1/Nrf2 Survival Pathway in Hippocampal HT22 Neuronal Cells

**DOI:** 10.3390/molecules27185813

**Published:** 2022-09-08

**Authors:** Wanchanok Nantacharoen, Seung Joon Baek, Waluga Plaingam, Somsri Charoenkiatkul, Tewin Tencomnao, Monruedee Sukprasansap

**Affiliations:** 1 Master Program in Clinical Biochemistry and Molecular Medicine, Department of Clinical Chemistry, Faculty of Allied Health Sciences, Chulalongkorn University, Bangkok 10330, Thailand; 2 Laboratory of Signal Transduction, Research Institute for Veterinary Science, College of Veterinary Medicine, Seoul National University, Gwanak-gu, Seoul 08826, Korea; 3 College of Oriental Medicine, Rangsit University, Pathum Thani 12000, Thailand; 4 Institute of Nutrition, Salaya Campus, Mahidol University, Nakhon Pathom 73170, Thailand; 5 Natural Products for Neuroprotection and Anti-Ageing (Neur-Age Natura) Research Unit, Faculty of Allied Health Sciences, Chulalongkorn University, Bangkok 10330, Thailand; 6 Department of Clinical Chemistry, Faculty of Allied Health Sciences, Chulalongkorn University, Bangkok 10330, Thailand; 7 Food Toxicology Unit, Institute of Nutrition, Salaya Campus, Mahidol University, Nakhon Pathom 73170, Thailand

**Keywords:** *Cleistocalyx nervosum* var. *paniala*, glutamate, molecular mechanisms, neuroprotection, neurotoxicity, neuron injury, oxidative stress, resveratrol, HT22 cells

## Abstract

Excessive glutamate neurotransmitters result in oxidative neurotoxicity, similar to neurodegeneration. An indigenous berry of Thailand, *Cleistocalyx nervosum* var. *paniala* (CNP), has been recognized for its robust antioxidants. We investigated the effects and mechanisms of CNP fruit extracts on antioxidant-related survival pathways against glutamate-induced neurotoxicity. The extract showed strong antioxidant capability and had high total phenolic and flavonoid contents, particularly resveratrol. Next, the protective effects of the CNP extract or resveratrol on the glutamate-induced neurotoxicity were examined in HT22 hippocampal cells. Our investigation showed that the pretreatment of cells with the CNP extract or resveratrol attenuated glutamate-induced neuronal death via suppression of apoptosis cascade by inhibiting the levels of cleaved- and pro-caspase-3 proteins. The CNP extract and resveratrol suppressed the intracellular ROS by increasing the mRNA expression level of antioxidant enzymes (SODs, GPx1, and CAT). We found that this extract and resveratrol significantly increased SIRT1 expression as a survival-related protein. Moreover, they also promoted the activity of the Nrf2 protein translocation into the nucleus and could bind to the promoter containing the antioxidant response element, inducing the expression of the downstream GPx1-antioxidant protein. Our data illustrate that the CNP extract and resveratrol inhibit apoptotic neuronal death via glutamate-induced oxidative neurotoxicity in HT22 cells through the activation of the SIRT1/Nrf2 survival mechanism.

## 1. Introduction

Nowadays, the ageing population is rapidly increasing worldwide, leading to a higher prevalence of various chronic and degenerative diseases, including Alzheimer’s disease [1,2]. The causes of Alzheimer’s disease include the accumulation of proteins in the brain [3,4], inflammation, and mitochondrial and endoplasmic reticulum stress dysfunctions, which are also associated with an increased oxidative stress. These are some of the main features underlying the progression of brain aging, leading to neurological damage in neurons, along with metabolic deficits, memory loss, and cognitive decline [5,6]. Moreover, prolonged and accumulated levels of neurotransmitters such as glutamate can influence and generate reactive oxygen species (ROS) and oxidative stress [7,8]. Excessive glutamate induces neurotoxicity and neuronal death in the central nervous system [9,10]. Subsequently, the overproduction of free radicals or oxidants can stimulate aberrant cellular function and also change the protein expression and function, leading to the occurrence of many neurodegenerative diseases. Therefore, a balance between oxidative stress and the antioxidant status is essential for neurological function [11,12]. *Cleistocalyx nervosum var. paniala* (CNP) belongs to the Myrtaceae family and is a native berry of Thailand. The ripe fruit has a sweet and astringent taste and presents a dark purple color. Previous studies have shown that CNP has high levels of anthocyanin, a high phenolic content, and has strong antioxidant activity [13,14,15]. The health benefits of CNP, which are supported by numerous studies, include antimutagenic, anticarcinogenic, and antigenotoxic activities [16,17,18]; antinephrotoxicity [19]; antineurotoxicity [15]; stress resistance and antiaging properties [19,20]; and increased human natural killer cell activity [21]. Interestingly, one bioactive compound found in these berries is resveratrol, which has powerful antioxidant activity [22,23]. Many scientific studies have found that resveratrol exhibits anti-inflammatory effects [23,24], increases plasma antioxidant ability levels, reduces lipid peroxidation in animals [25], enhances the activity of the catalase enzyme in mice [26], decreases oxidative stress protein biomarkers such as 8-hydroxyguanosine [27,28], and prevents neurotoxicity [29,30,31]. Resveratrol can stimulate and increase the expression of the SIRT1 gene and protein and is associated with cell survival and free radical scavenging by regulating the activation of the cellular antioxidant systems [32,33]. Based on the above information, however, the knowledge of the effects and cell-based molecular mechanisms of CNP on glutamate-induced oxidative neurotoxicity and neuronal death still remains limited. The present study investigates the effects and underlying mechanisms of CNP fruit extract against glutamate-induced oxidative damage and neurotoxicity. We further identify the upregulation of cellular antioxidant genes and survival protein markers induced by CNP fruit extract. The mouse hippocampal neuronal HT22 cell line is used as a cell model in our experiment.

## 2. Results

### 2.1. Antioxidant Capabilities and Resveratrol Content in CNP Extract

The antioxidant capability of the CNP extract was examined for its ability to scavenge free radicals, including the total phenolic and flavonoid contents, as exhibited in Table 1. Our results found that the scavenging activity of DPPH radicals by the CNP extract was 37.65 ± 4.72% with to 48.04 ± 4.72 mg ascorbic acid/g extract, while the quenching activity of ABTS radicals by the extract was 56.82 ± 0.86% with equal to 72.18 ± 0.93 mg ascorbic acid/g extract. Additionally, the contents of phenolic and flavonoid compounds in the CNP extract contained 383.07 ± 1.83 mg gallic acid equivalent/g extract and 43.71 ± 1.47 mg quercetin equivalent/g extract, respectively. Moreover, we also identified the resveratrol content in CNP via HPLC analysis. It was found to be 1.51 ± 0.07 mg/100 g DW, with 4.39% RSD in our CNP fruit extract. These investigations indicated that our CNP fruit extract showed high antioxidant capability and free radical scavenging activity, and we quantified the total phenolic and flavonoid compounds, including the constituent resveratrol, contained in this extract.

### 2.2. Cytotoxic Effects of CNP Extract on Viability of Cells in an Experiment Model of Glutamate-Induced Toxicity in HT22 Cells

**Figure 1:**
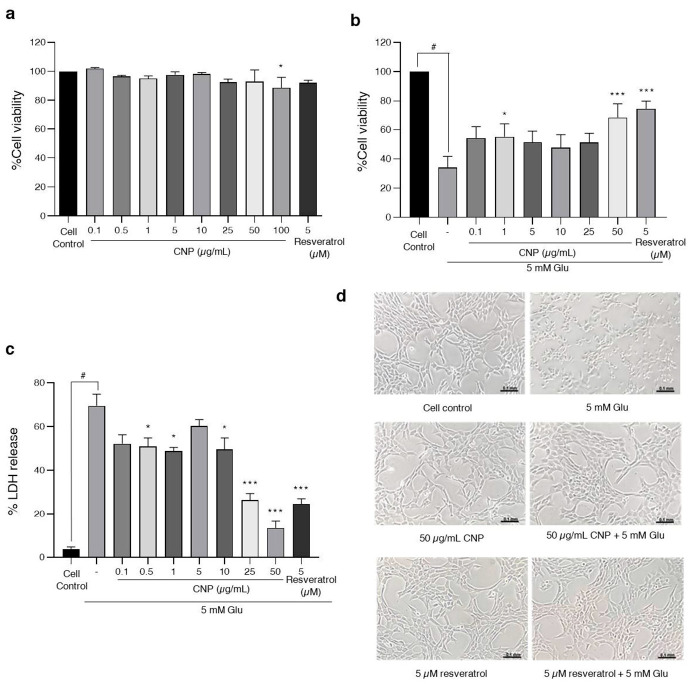
Effect of CNP extract on glutamate-induced cytotoxicity in HT22 cells. The cell viability was evaluated via MTT assay. (**a**) Cells were treated with CNP extract (0.1–100 µg/mL) for 24 h. (**b**) After treating the cells with CNP extract for 24 h, this was followed by 5 mM glutamate for 18 h. (**c**) The proportion of cell death with the percentage of LDH released was evaluated via LDH assay. (**d**) After the treatment, the morphology of HT22 cells was observed using a light microscope. Values are expressed as the means ± SD (*n* = 3). Note: *** *p* < 0.001 versus cell control, ^#^ *p* < 0.05 versus cell control, * p < 0.05 versus 5 mM glutamate-treated cells.

As examining the CNP extract might attenuate cell death in response to glutamate, we investigated the cytotoxic concentration of the CNP extract with and without glutamate. First, we determined the non-cytotoxic or cytotoxic concentrations of the CNP extract using MTT assay. HT22 cells were treated with the CNP extract at various concentrations (0.1–100 µg/mL) and with 5 µM resveratrol (positive control) for 24 h. Our results revealed that treating cells with 0.1–50 µg/mL of CNP extract was non-toxic, whereas the cytotoxic effect on HT22 cells was at 100 µg/mL compared with non-treated control cells (Figure 1a). Next, we investigated the protective effect of the CNP extract on the glutamate-induced toxicity in HT22 cells. The cells were pretreated with 0.1–100 µg/mL of CNP extract for 24 h prior to incubation with 5 mM glutamate for 18 h, then the cell viability was determined via MTT. Figure 1b shows that the glutamate treatment group significantly reduced the cell viability by approximately 32%. The treatment of the cells with 50 µg/mL of CNP extract significantly inhibited the glutamate-induced toxicity compared with the glutamate control group, with the highest protective effect of the CNP extract observed at 50 µg/mL, as shown in Figure 1b. Furthermore, we assessed the proportion of dead cells with the percentage of LDH release using an LDH-based cytotoxicity assay. The pretreatment of the cells with 0.5–50 µg/mL of CNP extract could significantly reduce cell death compared with the glutamate-treated group, while the glutamate treatment group significantly increased cell death by approximately 69%. This result was consistent with the cell viability result of the MTT assay; hence, the highest protective effect of the CNP extract was 50 µg/mL, as shown in Figure 1c. We also observed the cell morphology using a light microscope. The glutamate treatment caused toxic effects on cells and damaged the elongated shapes and structures of the cells. On the other hand, the pretreatment of cells with the CNP extract (50 µg/mL) and resveratrol (5 µM), which were used as a positive control in our model, improved the morphology of the cells and exhibited protective effects on the cells (Figure 1d). These results suggest that the pretreatment of cells with the CNP extract attenuates glutamate-induced neurotoxic effects in HT22 cells.

### 2.3. Effect of CNP Extract on Glutamate-Induced Apoptosis and Caspase-3 Protein Expression in HT22 Cells

**Figure 2:**
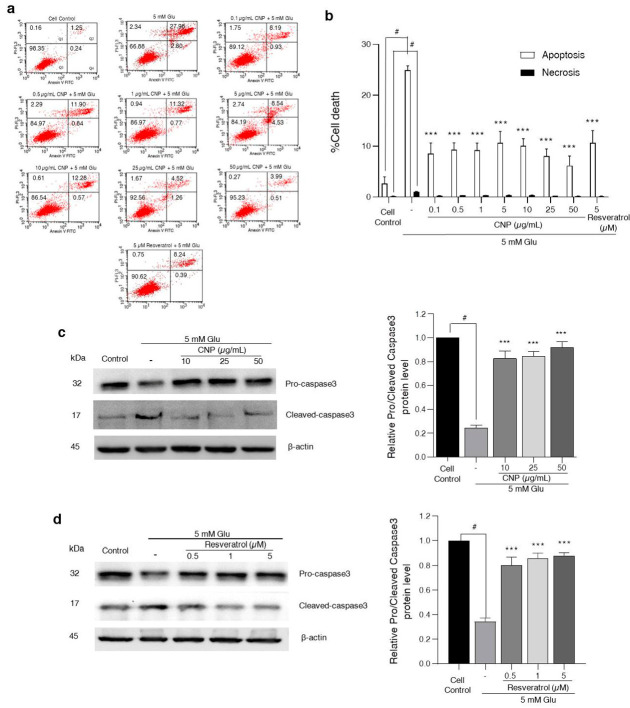
Effect of CNP extract on glutamate-induced apoptosis in HT22 cells. The cells were pretreated with CNP extract (0.1–50 µg/mL) for 24 h, followed by 5 mM glutamate for 18 h. After incubation, the HT22 cells were stained with Annexin V-FITC/PI probes, and (**a**) the numbers of cell deaths were analyzed via flow cytometry. The quarter sections represent the cells stages: Q1: necrosis; Q2: late apoptosis; Q3: live; Q4: early apoptosis. (**b**) The histogram represents the percentages of apoptotic and necrotic cells. For cells pretreated with CNP extract (**c**) or resveratrol (**d**) prior to glutamate incubation, the levels of caspase-3 protein expression were determined via Western blot, and histograms show relative of pro- or cleaved caspase-3 expression levels and normalized by β-actin. Values are expressed as the means ± SD (*n* = 3). Note: ^#^ *p* < 0.05 versus cell control, *** *p* < 0.001 versus 5 mM glutamate-treated cells.

To understand the characteristics of the cytotoxic effects of the CNP extract on mouse hippocampal neuronal cells, CNP-extract-treated HT22 cells were measured using the apoptosis assay via Annexin V-FITC and propidium iodide (PI) staining. This co-staining is widely used to determine the type of cell death. Annexin V is used to detect apoptotic cells, while PI is used to identify necrotic cells. Different concentrations (0.1–50 μg/mL) of CNP extract were added into the cell media and the cells were incubated for 24 h, followed by 5 mM glutamate for 18 h. Figure 2a,b show that the treatment of HT22 cells with 5 mM glutamate alone caused approximately 25% apoptosis and 1% necrosis, while the CNP-extract-treated cells, prior to glutamate incubation, could reduce the glutamate-induced apoptosis, with the highest protective effect observed at a concentration of 50 µg/mL. Moreover, the extract also suppressed the necrotic cells caused by glutamate. Likewise, the cells pretreated with 5 µM resveratrol significantly decreased both types of cell death induced by glutamate. Additionally, the apoptotic protein signal of HT22 neuronal cells was determined using a Western blot assay. We found that glutamate alone significantly activated the cleaved caspase-3 protein expression; meanwhile, it decreased the protein expression of pro-caspase-3 compared with the non-treated control group. Interestingly, the CNP extract and resveratrol significantly suppressed the level of cleaved caspase-3 protein expression compared to the glutamate-treated group (Figure 2c,d). This was consistent with the apoptotic results, as mentioned above. The CNP extract at 50 μg/mL showed higher efficiency than 5 μM resveratrol (positive control). Altogether, our results revealed that glutamate treatment has the potential to induce apoptosis and our CNP extract could inhibit the apoptotic cell death of HT22 neurons induced by glutamate.

### 2.4. Inhibition of Glutamate-Induced Intracellular ROS Generation via CNP Extract Treatment of HT22 Cells

**Figure 3:**
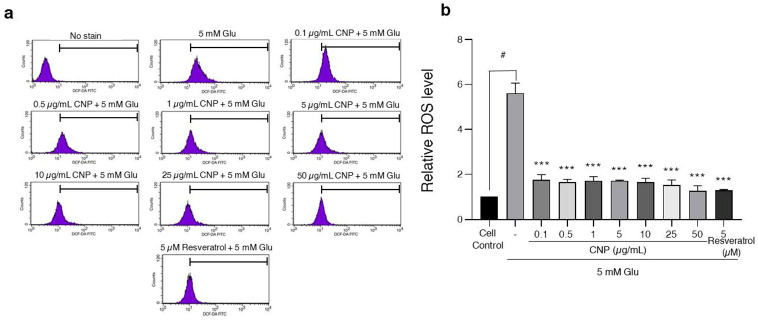
CNP extract treatment suppressed glutamate-induced ROS in HT22 cells. The cells were pretreated with CNP extract (0.1–50 µg/mL) or resveratrol (5 µM) for 24 h, followed by 5 mM glutamate 18 h. After this, the cells were incubated with 10 mM DCFH2-DA for 45 min at 37 °C. (**a**) The fluorescence intensity was measured via flow cytometry. (**b**) The histogram represents the relative ROS level of each treatment. Data are expressed as the relative ROS level compared to the non-treated control. The values are expressed as the means ± SD (*n* = 3). Note: ^#^ *p* < 0.05 versus cell control, *** *p* < 0.001 versus 5 mM glutamate-treated cells.

Further, to evaluate the glutamate-mediated neuronal apoptosis via the oxidative toxicity pathway and to determine the effects of the CNP extract or resveratrol on the glutamate-induced oxidative stress, the ROS levels of the HT22 cells were subjected to a DCFH2-DA fluorescent assay. The cells were pretreated with CNP extract at different concentrations (0.1–50 µg/mL) or resveratrol (5 µM) for 24 h, then followed by glutamate (5 mM) for 18 h. The results showed that the glutamate treatment significantly increased the intracellular ROS production by about 5-fold compared to the non-treated control group, whereas the cells pretreated with CNP extract saw significantly inhibited levels of intracellular ROS induced by glutamate (Figure 3a,b). The resveratrol treatment also reduced the ROS similarly to the CNP extract treatment. This result suggests that the CNP extract could suppress the intracellular ROS generation caused by glutamate in HT22 cells.

### 2.5. Effect of CNP Extract on the Cellular Antioxidant Enzymes in HT22 Cells

**Figure 4:**
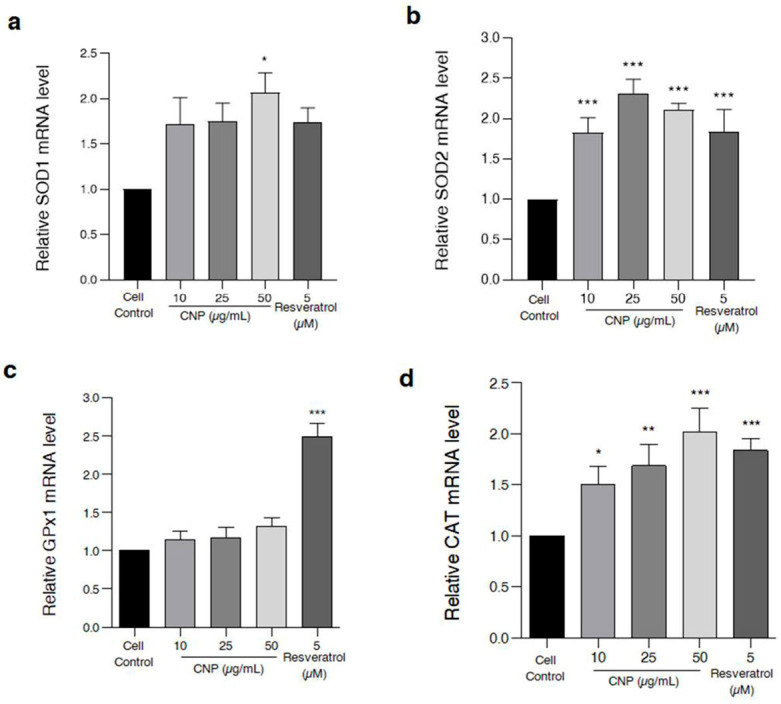
Effect of CNP extract on mRNA or gene expression levels of cellular antioxidant enzymes in HT22 cells. The cells were incubated with CNP extract (10–50 µg/mL) or 5 µM resveratrol as a positive control, and then the cells were harvested and the gene expression was measured. The levels of mRNA expression, such as (**a**) SOD1, (**b**) SOD2, (**c**) CAT, and (**d**) GPx1, were determined via quantitative real-time PCR. The data are shown as relative to mRNA expression and normalized by β-actin. Values are expressed as the means ± SD (*n* = 3). Note: * *p* < 0.05, ** *p* < 0.01, *** *p* < 0.001 versus non-treated control.

The major protective mechanism of ROS-mediated cellular damage is an antioxidant enzyme system including superoxide dismutase (SOD), catalase (CAT), and glutathione peroxidase1 (GPx1) [34,35,36,37]. Thus, in order to clarify the effect of the CNP extract on the expression of antioxidant genes in our HT22 hippocampal neuronal cells, the cells were incubated with CNP extract at different doses or with 5 µM resveratrol (used as positive control). The results showed that treatment of cells with CNP extract (10–50 µg/mL) could upregulate the mRNA expression levels of cellular antioxidant enzymes (SOD1, SOD2, CAT, and GPx1) (Figure 4). The cells treated with CNP extract at concentrations of 10–50 µg/mL tended to enhance the SOD1 expression, especially at the highest concentration (50 µg/mL), which significantly provoked the gene level of SOD1 compared with the cell control. Furthermore, the treatment of cells with the extract (10–50 µg/mL) significantly increased the mRNA expression levels of SOD2 and CAT compared to the cell control group, while the treated cells with the extract slightly enhanced the GPx1 mRNA levels. Additionally, the treatment of the cells with resveratrol was shown to elevate the gene expression levels of these antioxidant enzymes. Our results demonstrate that treatment with CNP extract clearly upregulates the mRNA or gene expression of cellular antioxidant enzymes, and these results also suggest that CNP extract acts as a potent neuroprotective antioxidant against oxidative toxicity in this hippocampal neuronal HT22 cell model.

### 2.6. Effect of CNP Extract on SIRT1 Survival Protein Expression

**Figure 5:**
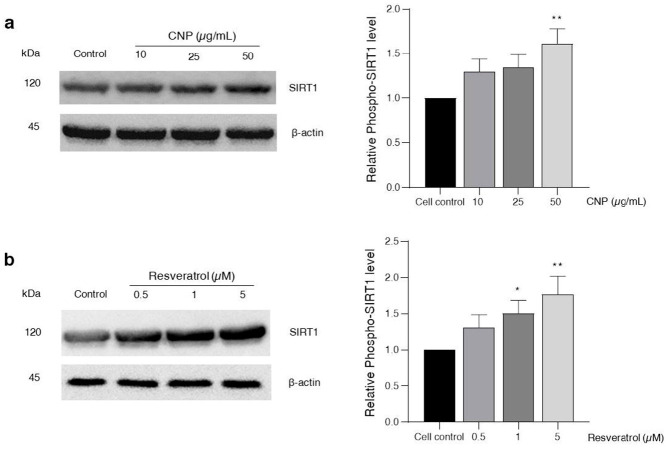
Effect of CNP extract on SIRT1 protein expression in HT22 cells. Cells were incubated with (**a**) CNP extract (10–50 μg/mL) or (**b**) resveratrol (0.5–5 μM) for 24 h. The levels of protein expression were determined by Western blot analysis. The protein expressions are shown as relative to the SIRT1 level and normalized by β-actin. The data are represented as the means ± SD (*n* = 3). Note: * *p* < 0.05, ** *p* < 0.01 versus non-treated control.

The SIRT1 protein mainly regulates the cellular survival pathway and the group of stress response genes. It plays a key role in extending life by stimulating the many antioxidant genes and proteins [38,39]. To further determine the effect of the CNP extract on SIRT1 protein expression, which is related to the cell survival pathway, the HT22 cells were treated with CNP extract at 10–50 μg/mL for 24 h. The results demonstrate that all concentrations of CNP extract were able to increase the expression of the SIRT1 protein, and that the CNP extract at concentrations of 50 μg/mL in particular could significantly increase the levels of this protein compared with the non-treated control group (Figure 5a). In addition, we also assessed the treatment of cells with three high-efficiency doses of resveratrol (0.5–5 μM). All of the resveratrol concentrations enhanced the SIRT1 expression, particularly with 1 and 5 μM of resveratrol, compared with the non-treated control group (Figure 5b). Our results suggest that the CNP extract can promote the SIRT1 survival protein expression of the hippocampal neuronal cells, and this mechanism has the potential to prevent oxidative glutamate toxicity.

### 2.7. Effect of CNP Extract on Nrf2 Protein Translocation

**Figure 6:**
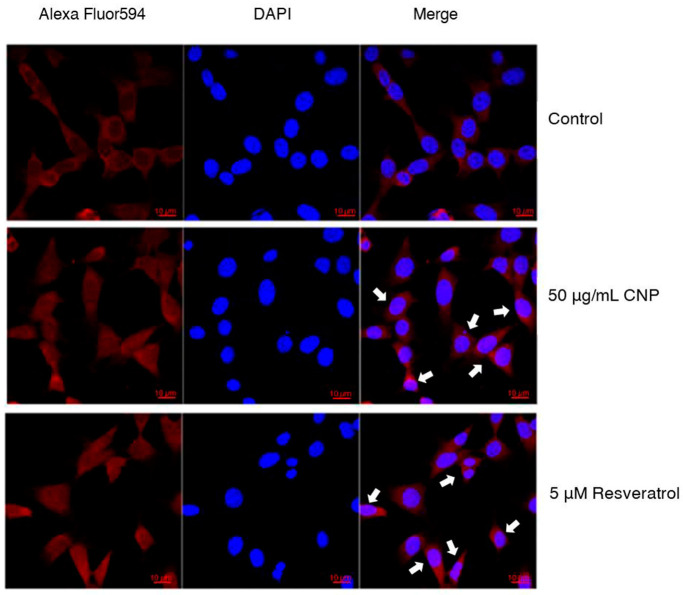
Effect of CNP extract and resveratrol on the translocation of Nrf2 transcription factor into the nucleus. HT22 cells were treated with 50 μg/mL CNP extract or 5 μM resveratrol for 24 h. The cells were assessed via fluorescent staining with Alexa Fluor 594 (red color) to indicate the Nrf2 protein. DAPI (blue color) fluorescent nuclear staining was performed. Then, the cells were observed under confocal microscopy (scale bar is 10 μm).

In the results above, we demonstrated the ability of the CNP extract to reduce ROS levels and apoptotic cell death, and also to stimulate antioxidant genes in our studies. Next, we investigated whether the CNP extract treatment enhances the activity of Nrf2 transcription factor protein. This is the key transcription factor that regulates the antioxidant response in neurons [40,41]. Therefore, to verify the effect of the CNP extract on Nrf2 protein translocation into the nucleus, we examined the protein translocation via immunofluorescence assay and observed it under confocal microscopy. The highest protective effect of the CNP extract at 50 μg/mL and of resveratrol at 5 μM were selected for investigation in this experiment. The HT22 cells were treated with 50 μg/mL CNP extract or 5 μM resveratrol for 24 h. We used the Alexa Fluor 594 fluorescent dye, which is a secondary antibody conjugated to the Nrf2 primary antibody, to indicate the Nrf2 protein. Our results showed that the CNP extract and resveratrol could obviously translocate the Nrf2 protein from the cytoplasm into the nucleus more than the cell control group, as shown in Figure 6. This finding indicates that the CNP extract and resveratrol appear to stimulate the activity of the Nrf2 transcription factor protein, which is associated with regulating cellular survival and antioxidant response in our HT22 cell model.

### 2.8. Effect of CNP Extract on the Ability to Bind the Antioxidant Response Element (ARE) Promoter Element

**Figure 7:**
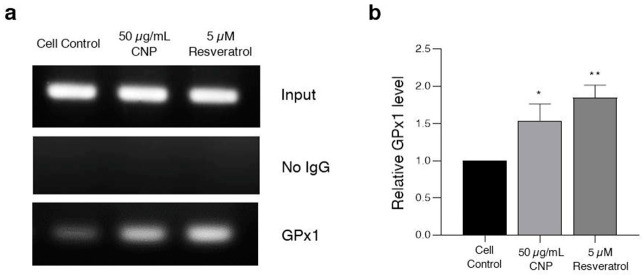
Effect of the CNP extract and resveratrol on the ability to bind the ARE promotor, inducing the expression of the downstream GPx1 antioxidant protein. HT22 cells were pretreated with 50 μg/mL CNP extract or 5 μM resveratrol for 24 h. The capability for protein–DNA interactions was detected via chromatin immunoprecipitation (ChIP) assay. (**a**) The product from the ChIP was replicated via PCR. (**b**) The data are shown as relative to the Gpx1 expression level and normalized by input (internal control). Values are represented as the means ± SD (*n* = 3). Note: * *p* < 0.05, ** *p* < 0.01 versus non-treated control.

To confirm the activity of Nrf2 binding to the specific ARE promoter to induce expression of the downstream antioxidant protein in response to the CNP extract and resveratrol, the interaction between the protein and DNA in the cell was evaluated via ChIP assay. Thus, based on our HT22 cell model, the cells are involved in the cellular antioxidant glutathione, which is used as a substrate for the synthesis of GPx1. It is one of the downstream targets of the Nrf2 protein. Additionally, GPx1 was chosen for this investigation. Interestingly, we found that cells treated with 50 μg/mL CNP extract or 5 μM resveratrol showed increased Nrf2 protein function in regard to binding on the ARE position on the DNA strand compared with the non-treated control group, as shown in Figure 7a. Moreover, the cells treated with both the CNP extract and resveratrol displayed significantly enhanced expression of downstream GPx1 antioxidant enzyme (Figure 7b). These findings suggest that the CNP extract and resveratrol can bind the ARE promoter element and obviously activate the targeted downstream Nrf2 protein.

## 3. Discussion

The nervous system has various neurotransmitters; one of them is glutamate, which can induce neurotoxicity, called excitotoxicity, through binding its receptors. One of the receptor pathways is mediated by the ionotropic glutamate receptors. If the glutamate uptake via these receptors is saturated or dysfunctional due to the excessive production and accumulation of glutamate in the neurons, it can lead to the generation of intracellular ROS and oxidative stress in the neurons [42]. Excessive ROS and oxidative stress plays a crucial role in the neurotoxic effects that cause neuronal cell damage and apoptosis through interactions with membranes and the induction of lipid peroxidation, oxidizing RNA and DNA to interrupt transcription and protein oxidation, and are involved in several neurodegenerative disorders [43,44,45]. In addition, glutamate can get into neuronal cells via another pathway, the glutamate/cystine antiporter pathway, which is a non-receptor-dependent way to produce ROS via excessive glutamate, such as that present in the murine hippocampal neuronal HT22 cell model [15,46]. The excessive extracellular glutamate can inhibit the cystine uptake via interaction with the glutamate/cystine antiporter pathway [46,47]. Consequently, this event results in the diminution of intracellular glutathione (GSH) levels and the accumulation of ROS and oxidative stress, and finally neuronal cell death [42]. These correlative effects between oxidative stress and glutamate toxicity have been reported in various neuronal cell lines and primary neurons [48,49]. For example, the HT22 cell line is extensively used as an in vitro model for investigating glutamate-induced oxidative stress pathways, contributing to neuronal dysfunction and cell death. This cell line has been shown in many reports to be involved in hippocampal neuron function [15,34,50,51,52].

Normally, the cellular or endogenous antioxidant system eliminates the intracellular free radicals or ROS, but if the antioxidants number less than the free radicals, they induce cell damage, dysfunction, and death [53,54]. Cellular antioxidant enzymes (such as Cu/Zn-SOD or SOD1, Mn-SOD or SOD2, GPx1, and CAT) serve to dispose of the free radicals [55]. The expression and activity of these antioxidant enzymes can be increased by numerous phytochemicals and bioactive compounds [51,52,56]. For example, a group of flavonoid substances including anthocyanins and phenolic compounds such as resveratrol have been studied, which can pass the blood–brain barrier to protect affected nerve cells [57,58]. Among the natural polyphenols, resveratrol (trans-3,5,4′-trihydroxystilbene) is well known as one of the neuroprotective antioxidants [59,60,61,62]. It belongs to a class of polyphenolic compounds found in berries such as grapes, mulberries, and other berries [63,64]. Our present study demonstrates that the CNP berry fruit extract shows high levels of phenolic compounds and flavonoids. The CNP extract can certainly scavenge free radicals in both ABTS and DPPH assays (Table 1), indicating the high antioxidant capabilities of this fruit, which is consistent with several studies [14,15,65]. Furthermore, we also identified resveratrol as a distinctive bioactive compound in CNP berry extract via HPLC analysis, which is presented for the first time in this paper. Thus, this berry extract has the potential to be used for pretreatment in a model of HT22 hippocampal neuronal cell injury induced by glutamate.

Subsequently, we investigated the effects and underlying mechanisms of the CNP berry extract on glutamate-induced neurotoxicity. Initially, we observed the non-toxic concentrations of CNP extract in various concentrations. Moreover, our results reveal that the CNP extract in non-toxic doses (0.1–50 µg/mL) can suppress the oxidative damage induced by glutamate (Figure 1a–d). We confirmed that the CNP extract and resveratrol were able to increase the cell viability and decrease apoptotic cell death in our cell model using Annexin V-FITC/PI double staining (Figure 2a,b). Glutamate treatment alone significantly induced toxicity and activated apoptotic cell death, suggesting that glutamate causes an essential mechanism mediating apoptosis in our HT22 cells. Additionally, we investigated the expression of an apoptotic protein effector, namely cleaved caspase-3, compared with the non-treated control group. Remarkably, the CNP extract and resveratrol could inhibit the apoptotic cleaved caspase-3 protein expression of HT22 neuronal cells (Figure 2c,d), which linked with the apoptotic results mentioned above. In addition, pretreatment of the cells with CNP extract or resveratrol significantly reduced the glutamate-induced intracellular ROS generation (Figure 3), indicating that glutamate induces neuronal apoptosis via the oxidative toxicity pathway in the HT22 cell model. These neuroprotective effects are possibly involved in the powerful antioxidant capabilities of the CNP berry extract and the resveratrol-bioactive substance, as presented in this fruit. Likewise, it has been shown that the CNP extract reduced glutamate-induced ROS accumulation and cell death in neuronal cells [15]. The bioactive compounds present in the CNP extract may act synergistically to suppress oxidative neurotoxicity observed in the present study.

For the mechanistic approach in neuroprotection, our experiment furthermore exhibited that the reduced glutamate-mediated oxidative stress and apoptosis was due to the ability of the CNP extract to upregulate cellular antioxidant enzyme expression in HT22 cells. We found that pretreating cells with CNP extract certainly enhanced the gene expression levels of antioxidant enzymes, including SOD1, SOD2, CAT, and GPx1 (Figure 4). These antioxidant enzymes are the main endogenous antioxidant enzymes that play an important role in the balance between pro-oxidant or free radicals and antioxidant activities in the neurons [66,67,68]. The mechanisms of action of SIRT1 and Nrf2 control the expression of oxidative stress protection biomarkers such as SODs, CAT, GPx1, and glutathione. This pathway has an essential role in survival, extending life by upregulating numerous antioxidant genes and proteins [38,39,69]. In the present study, we found that the CNP extract distinctly upregulated the expression of the SIRT1 protein (Figure 5a), as well as resveratrol (Figure 5b) in our cell model, indicating that the CNP extract and its bioactive compound can promote the SIRT1 survival protein expression of the hippocampal neuronal cells. Therefore, this mechanism has the possibility to prevent oxidative glutamate toxicity. Previously, resveratrol has been reported to show powerful antioxidant properties in both direct and indirect cellular effects, and it shows the best effect in terms of increasing a specific survival protein, namely SIRT1, in many cells, including neuronal cells [70,71,72]. In addition, it is well known that Nrf2 is the key transcription factor that regulates antioxidant response genes and proteins in neurons [73,74]. Consequently, we observed the translocation of the Nrf2 protein and also determined the binding of Nrf2 to the ARE promoter element site on the DNA strand. Our investigation showed that the Nrf2 protein in the cytoplasm could translocate into the nucleus (Figure 6) in response to CNP extract or resveratrol treatment. Moreover, we found that the CNP extract and resveratrol were able to directly bind Nrf2 transcription proteins to the ARE promoter site on DNA strands in the nucleus, resulting in the expression of targets downstream, such as the GPx1-antioxidant protein (Figure 7), in HT22 cells. Several reports have shown that SIRT1 is an important protein that connects to the activation of the Nrf2/ARE signaling pathway, resulting in neuroprotection [75,76]. Additionally, many studies have revealed that SIRT1 can increase the Nrf2 protein level and improve oxidative stress via natural bioactive compounds from berry fruits such as anthocyanins and resveratrol [51,77]. Thus, the Sirt1/Nrf2 pathway is considered to be a promising target for protecting neuronal cells from oxidative damage and death. Our findings indicated that the CNP extract and resveratrol-bioactive substances can effectively enhance the SIRT1 protein expression and stimulate the activity of the Nrf2 transcription factor protein, which is associated with regulating cellular survival and antioxidant response, leading to the reduction in intracellular ROS in our HT22 cell model.

## 4. Materials and Methods

### 4.1. Chemicals and Reagents

The 3,4′,5-trihydroxy-trans-stilbene (resveratrol; purity ≥ 99%), L-glutamic acid, Dulbecco’s modified Eagle’s medium (DMEM), and fetal bovine serum (FBS) were purchased form Sigma-Aldrich (St. Louis, MO, USA). The 3-(4,5-dimetylthiazol-2-yl)- 2,5-diphenyltetra-zoliumbromide (MTT) was purchased from Bio Basic (Markham, ON, Canada). The 2′,7′-dichlorodihydrofluorescein diacetate (H_2_DCFDA) was obtained from Molecular Probes (Eugene, OR, USA). The Trizol reagent was purchased from Invitrogen (Carlsbad, CA, USA). The penicillin–streptomycin solution was purchased from Gibco (Waltham, MA, USA). The annexin V-FITC apoptosis detection kit was purchased from BioLegend (San Diego, CA, USA). The RT Pre-Mix and qPCR Master Mix solutions were obtained from Bioneer (Daejeon, South Korea). The chromatin immunoprecipitation (ChIP) assay kit was purchased from Merck (Kenilworth, NJ, USA).

### 4.2. CNP Collection and Extraction

The ripe CNP fruit samples were collected during July–August 2018, from the cultural practice area in Lampang province, Thailand, initiated by the Plant Genetic Conservation Project under the Royal Initiation of Her Royal Highness Princess Maha Chakri Sirindhorn (RSPG). This plant was identified and authenticated, and the scientific name was confirmed by Assistant Professor Dr. Thaya Jenjittikul, Department of Plant Science, Faculty of Science, Mahidol University, Thailand. The voucher specimen was deposited at Suan Luang Rama IX Herbarium (9428), Bangkok, Thailand. The ripe CNP fruit samples were destalked and washed with deionized water. The pulp was separated from the seed, weighed and dried with a freeze dryer, then ground to a fine powder. The CNP powder was extracted using 95% ethanol at a solvent ratio of 1:10 using a Soxhlet extractor. Then, the extract was evaporated, the solvent was removed using a vacuum rotary evaporator, and the extract was lyophilized [15,78]. The yield of the extract was 39.85%. This stock of crude extract was dissolved in 100% DMSO to obtain a sample concentration of 100 mg/mL, filtrated through a 0.2 μm sterile filter, and stored at −20 °C in darkness.

### 4.3. The 2,2-Diphenyl-1-Picrylhydrazyl (DPPH) Assay

The total free radical scavenging capability was detected using the DPPH radical scavenging method as reported by Sukprasansap et al. [15]. The DPPH working solution was dissolved in methanol and ascorbic acid was used as a standard antioxidant. The 20 μL of CNP extract was added in a 96-well plate followed by 180 μL of DPPH working solution. After incubation in the dark for 30 min at room temperature, the absorbance was measured at 517 nm using a microplate reader. The results were calculated and are presented as the percentages of scavenging activity [15].

### 4.4. The 2,2’-Azinobis-(3-Ethylbenzothiazoline-6-Sulfonic Acid) (ABTS) Assay

The ABTS radical cation decolorization assay was used to detect the scavenging effect of the CNP extract. The ABTS working solution was mixed with 7 mM ABTS and 2.45 mM potassium persulfate (ratio 1:1.5), then stored in the dark for 16–18 h at 4 °C. Next, ABTS ^+^ was diluted with ethanol to 0.7 ± 0.02 at 734 nm. The CNP extract (20 μL) and ABTS working solution (180 μL) was added into each well. Ascorbic acid was used as a standard antioxidant. Next, the mixture was incubated in the dark for 45 min at room temperature. The plate was read in a microplate reader at 734 nm and the data are expressed as percentages of scavenging activity.

### 4.5. Total Phenolic Content

The total phenolic content of the extract was determined quantitatively using the Folin–Ciocalteu colorimetric method. Briefly, gallic acid was used for standard curve determination. The 10% Folin–Ciocalteau reagent and 0.1 M of sodium carbonate solution (Na_2_CO_3_) were transferred (50 μL) into a 96-well microplate. After mixing with the CNP extract, the reaction mixture was dissolved with methanol to 1 mg/mL 50 μL, and then incubated at room temperature for 60 min in darkness. The absorbance of the solution was then measured at a wavelength of 760 nm using a microplate reader. The total phenolic compound value was calculated with standard curves of gallic acid and expressed as milligrams of gallic acid equivalent per gram of extract (mg gallic acid equivalent/g extract).

### 4.6. Total Flavonoid Content

The total flavonoid content was measured via aluminum chloride colorimetric assay. Quercetin was used for the standard curve. The CNP extract was dissolved in methanol at a concentration of 1 mg/mL and then the 50 μL of solution was transferred into a 96-well plate. Next, 10 μL of 10% aluminum chloride (AlCl_3_) and 10 μL of 1 M sodium acetate (NaOAc) were added into each well. The 150 μL of ethanol solution was mixed in a 96-well plate. The reaction mixture was incubated in darkness at room temperature for 40 min and the absorbance was measured using a microplate reader at 415 nm. The total flavonoid content was compared with the quercetin standard curve and presented in milligrams of quercetin equivalent per gram of extract (mg quercetin equivalent/g extract).

### 4.7. Determination of Resveratrol in CNP Extract by HPLC Method

The resveratrol content of the CNP extract was measured via high-performance liquid chromatography with diode array detection (HPLC-DAD) using the SHIMADZU LC-10 series system (Shimadzu Scientific Instruments, MA, USA) and a Zorbax Eclipse XDB-C18 column (4.6 × 150 mm). The HPLC gradient analysis was performed according to the method reported by Sukprasansap and colleagues [15]. Briefly, the mobile phases were phase A (2% acetic acid in water) and phase B (methanol). The elution was delivered at a flow rate of 1.0 mL/min. The gradient elution was conducted at 90% A and 10% B (0−40 min), 50% A and 50% B (40−45 min), and 90% A and 10% B (45−60 min). The sample (20 µL) was directly injected into the column and controlled by heating at 35 °C. The resveratrol was measured at 306 nm using UV-DAD. The peak identity was confirmed by comparing retention times and the spectral absorption with pure resveratrol (purity ≥ 99%, Sigma Chemical Co., St. Louis, MO, USA), then quantified by comparing the results with the standard curve. The resveratrol content was indicated as mg/100 g dry weight (DW).

### 4.8. Cell Culture

The murine hippocampal neuronal HT22 cells were generously donated by Prof. David Schubert (The Salk Institute, San Diego, CA, USA). They were cultured in Dulbecco’s modified Eagle’s Medium (DMEM) supplemented with 10% heat-inactivated fetal bovine serum, penicillin G (100 units/mL), and streptomycin (100 µg/mL) under 5% CO_2_ in a humidified atmosphere at 37 °C. The adhered cells were grown to 70–80% confluence.

### 4.9. Cell Viability Assay

The viability of the cells after the treatment with the sample and glutamate were evaluated via 3-(4, 5-dimethylthiazol-2-yl)-2,5diphenyltetrazolium bromide (MTT)-based colorimetric assay. Briefly, the HT22 cells were seeded in a 96-well flat-bottom culture plate at a density of 3000 cells/well overnight and treated with glutamate at 1, 3, 5, and 10 mM for 18 h to select the appropriate concentration to use. Then, the cells were treated with CNP at 0.1, 0.5, 1, 5, 10, 25, and 50 µg/mL in each well for 24 h. After incubation, the cells were added to each well with MTT solution at 37 °C in a humidified 5% CO_2_ atmosphere for 3 h. Next, the supernatant was removed and the formazan crystals were solubilized in 100 µL of DMSO. The absorbance of each well was measured at 550 nm using a microplate reader (Perkin Elmer, Waltham, MA, USA). The percentage of cell viability was expressed relative to the control cells.

### 4.10. Lactase Dehydrogenase (LDH) Assay

This method is a colorimetric assay that determines the cellular cytotoxicity and the effect of cell death. It measures the amount of LDH enzyme that is secreted into the culture medium upon damage to the plasma membrane. The standard protocol reported here was performed according to the manufacturer’s instructions using a CytoTox 96^®^ non-radioactive cytotoxicity assay kit. Briefly, the HT22 cells were seeded in 96-well flat-bottom culture plates at a density of 3000 cells/well overnight before being treated with different concentrations of sample for 24 h, then exposed to 5 mM glutamate. After 18 h, the media were transferred to a new microplate and 50 µL of substrate mix was added from the kit. The plate was incubated in darkness at room temperature for 30 min before adding 50 µL of the stop solution. The absorbance was measured at 490 nm using a microplate reader. The results were analyzed by calculating the LDH release activity normalized to the maximum LDH release control. The data were reported as percentages of LDH release.

### 4.11. ROS Detection Assay

The intracellular ROS production was quantified using the DCFH_2_-DA fluorescent probe. Briefly, the cells at a density of 3.5 × 10^4^ cells were seeded on 12-well plates and then pretreated with CNP extract at various concentrations for 24 h. Next, the treatment cells were exposed to 5 mM glutamate. After 18 h of incubation, the cells were loaded with 10 µM DCFH_2_-DA for 45 min at 37 °C. Then, the cells were washed, trypsinized, and resuspended in buffer. The fluorescent intensity was analyzed via flow cytometry (FACSCalibur, BD Biosciences, San Jose, CA, USA) with excitation and emission wavelengths at 495 and 529 nm, respectively.

### 4.12. Apoptosis Assay

The apoptotic cell death was quantified via flow cytometry using the fluorescence isothiocyanate (FITC)-annexin V apoptosis detection kit with propidium iodide (PI) (BioLegend, San Diego, CA, USA) according to the manufacturer’s protocol. Briefly, the cells were seeded at a density of 3.5 × 10^4^ cells on a 12-well plate, grown overnight, and treated with varying concentrations of CNP extract for 24 h. Next, the treated cells were exposed to 5 mM glutamate for 18 h. The cells were harvested at the end of the treatment. The cells were washed using phosphate-buffered saline (PBS) and resuspended in binding buffer before staining with FITC-conjugated the annexin V and PI solution for 15 min in the dark. The apoptotic cells were determined via flow cytometry (FACSCalibur, BD Biosciences, San Jose, CA, USA) with excitation and emission wavelengths at 488 and 518 nm, respectively.

### 4.13. Gene Expression Analysis by Quantitative Real-Time PCR Analysis (qRT-PCR)

The total RNA was extracted from the treated cells using Trizol reagent (Invitrogen, Carlsbad, CA, USA) according to the manufacturer’s instructions. The concentration and quantity of the RNA were determined using Nanodrop (Thermo Scientific, Waltham, MA, USA) with absorbance at 260 nm and 280 nm. The cDNA was synthesized using 1 µg of total RNA using AccuPower RT Premix (Bioneer, Daejeon, South Korea) and oligo (dT) 17 primer. The qRT-PCR was conducted with the Exicycler Real-Time Quantitative Thermal Block (Bioneer, Daedeok-gu, Korea). The amplifications of sDNA were detected using Green Star PCR Master Mix (Bioneer, Daejeon, Republic of Korea) and specific primers for SOD1 (forward: 5′-CAGGACCTCATTTTAATCCTCAC-3′; reverse: 5′-CCCAGGTCTCCAACATGC-3′), SOD2 (forward: 5′-CTGGACAAACCTGAGCCCTA-3′; reverse: 5′-TGATAGCCTCCAGCAACTCTC-3′), CAT (forward: 5′-CAGCGACCAGATGAAGCA-3′; reverse: 5′-CTCCGGTGGTCAGGACAT-3′), and GPx1 (forward: 5′-ACAGTCCACCGTGTATGCCTTC-3′; reverse: 5′-CTCTTCATTCTTGCCATTCTCCTG-3′), as well as the normalized primer β-actin as an endogenous control (forward: 5′-GGCTGTATTCCCCTCCATCG-3′; reverse: 5′-CCAGTTGGTAACAATGCCATGT-3′) [15,34]. All gene amplifications were performed with the following thermal cycling conditions: 95 °C for 15 min, followed by 45 cycles of denaturation at 95 °C for 15 s and primer annealing/extension at 55 °C for 30 s. A melting curve analysis was performed to determine the primer specificity. The relative expression data were normalized to β-actin gene expression and the levels of gene expression were analyzed using the 2^−ΔΔCT^ method.

### 4.14. Western Blot Analysis

The specific treated cells were harvested and the cell lysate was prepared according to the previous protocol described by Sukprasansap et al. [15]. Briefly, the cells were lysed in the lysis buffer containing 1X RIPA cell lysis buffer with EDTA, 1% sodium orthovanadate, 2% phenylmethylsulfonyl fluoride, and 1% aprotinin for 10 min on ice, then the cells were lysed via sonication (Bandelin, Berlin, Germany). The protein concentrations were quantified via Bradford assay (Bio-Rad, CA, USA). An equal amount of protein was denatured by heating in Laemmli loading buffer at 95 °C and separated on 10% SDS–polyacrylamide gel, then transferred to nitrocellulose membranes (GE Healthcare, Camberley, UK). After blocking for 1 h with 5% skim milk in TBST (25 mM Tris-HCl, pH 7.5, 125 mM NaCl, and 0.05% Tween 20), the membranes were incubated with primary antibodies specific for SIRT1 (1:1000; Cell Signaling), caspase-3 (1:1000; Santa Cruz, Heidelberg, Germany), cleaved caspase-3 (1:1000; Santa Cruz, Heidelberg, Germany), and β-actin (1:2000; Santa Cruz, Heidelberg, Germany) at 4 °C overnight. The membranes were washed 3 times with TBST for 15 min and subsequently with anti-rabbit IgG HRP-linked antibody (1:5000; Thermo scientific, Waltham, MA, USA) at room temperature for 1 h. The specific protein bands were visualized with enhanced chemiluminescence using the ECL Western blotting substrate (Thermo Scientific, Waltham, MA, USA) using a UVITEC Cambridge machine (Alliance, Cambridge, UK) and quantified using Image J software, then normalized with β-actin.

### 4.15. Immunofluorescence Microscopy Assay

The treated cells were fixed with cold 4% paraformaldehyde for 10 min, washed 3 times with PBS, added to 0.2% Triton X-100 in PBS for 10 min, and blocked with 5% bovine serum albumin (BSA) for 1 h. Next, the cells were incubated with primary antibodies against Nrf2 (1:500; Santa Cruz, Heidelberg, Germany) overnight at 4 °C, followed by incubation with a secondary antibody (Alexa Fluor 594-conjugated goat anti-rabbit (1:2000), Invitrogen, Waltham, MA, USA) for 1 h at room temp. The nucleus was stained with 4, 6-diamidino-2-phenylindole (DAPI) for 10 min. The images were captured using a confocal microscope (ZEISS, Oberkochen, Germany).

### 4.16. Chromatin Immunoprecipitation (ChIP) Assay

After the treatment of HT22 cells with CNP extract, they were fixed with 37% formaldehyde to cross-link the DNA and proteins, then they were lysed in SDS lysis buffer and the sonication technique used to shear the DNA. Next, the short-strand DNA was analyzed according to the protocol from the chromatin immunoprecipitation assay kit (Merck, Burlington, MA, USA) protocol. Briefly, the supernatant was diluted 10 times with ChIP dilution buffer and divided for purification and PCR measurements, then was used as an input control (internal control). The diluted cell supernatant was administered with 75 µL of Protein A Agarose/Salmon Sperm DNA for 30 min at 4 °C in order to reduce the non-specific background. After this, the supernatant fraction was incubated with Nrf2 antibody (1:500; Santa Cruz, Heidelberg, Germany) overnight at 4 °C with rotation. Next, Protein A Agarose/Salmon Sperm DNA was applied to precipitate the DNA/Nrf2 antibody complex and the supernatant was removed. The pellet was washed with the buffers and rinsed with an elution buffer to precipitate the beat. The supernatant containing the DNA/Nrf2 antibody complex was added to 5 M NaCl, 0.5 M EDTA, 1 M Tris-HCl, and 10 mg/mL proteinase K, according to the manual, then the remaining DNA was purified with phenol–chloroform. The purified DNA was quantitatively analyzed via PCR. A specific primer of the GPx1-antioxidant enzyme gene (forward: 5’-CGGGACCCTGAGACTTAGA-3 ′; reverse: 5’-CGAGCAGCACACACATACTG-3’) was used in this experiment. This gene enters the ARE promoter position, where the Nrf2 transcription factor protein binds on the DNA for the regulation and signaling of the downstream target genes. The targeted DNA fragments were separated by agarose gel electrophoresis and photographed using a UVITEC Cambridge machine (Alliance, Cambridge, UK). The density of the targeted bands was quantified using Image J software, then normalized with the internal control group.

### 4.17. Statistical Analysis

The data are expressed as the means ± standard deviation (SD) from at least three independent experiments, and we performed a one-way ANOVA followed by the Tukey post-test at a significance level of *p* < 0.05. The data were analyzed using Graphpad Prism Software (version 5 for Windows; Graphpad software Inc.: San Diego, CA, USA, 2010).

## 5. Conclusions

**Figure 8:**
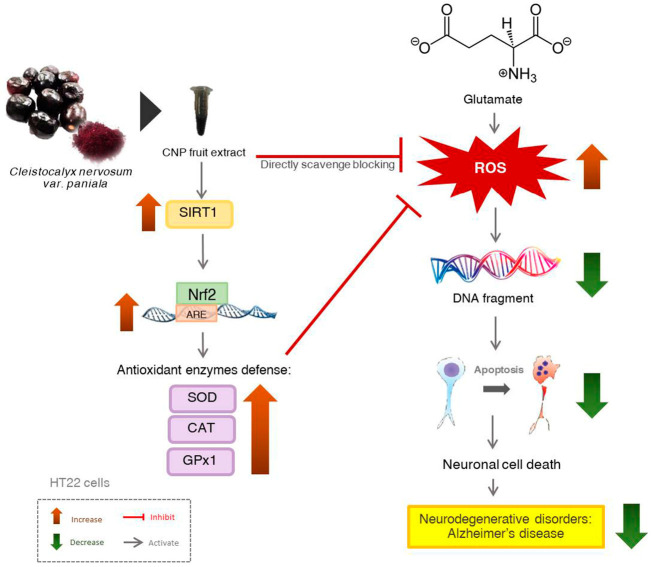
Schematic overview of the proposed mechanism of CNP protection against glutamate-induced neuronal death in HT22 hippocampal neuronal cells. CNP can directly scavenge and suppress the intracellular ROS and also activate the cellular antioxidant enzyme genes via SIRT1/Nrf2 survival pathway, resulting in the inhibition of neurotoxicity and apoptotic neuronal death caused by glutamate in HT22 cells.

This study demonstrates the antioxidant neuroprotective action of the CNP extract in murine hippocampal neuronal HT22 cells. Our results illustrate that pretreatment of HT22 cells with the CNP extract and resveratrol-bioactive substances attenuate glutamate-induced oxidative toxicity and apoptosis by repressing the intracellular ROS elevation and promoting endogenous antioxidant enzymes, namely SOD1, SOD2, CAT, and GPx1 via the SIRT1/Nrf2 survival pathway, as shown in Figure 8. Taken together, this information suggests that the CNP extract and resveratrol act as potent antioxidant and neuroprotective agents and could be beneficial in preventing age-related neurological disorders. In future studies, this fruit warrants investigation in both animal and human experiments. It may be further developed as a dietary neuroprotectant product that has the effect of preventing neurodegenerative diseases, delaying the severity of the disease. Furthermore, this study may provide the clue to develop a novel drug for the treatment of neurodegenerative diseases such as Alzheimer’s disease.

## Figures and Tables

**Table 1:** Antioxidant capabilities and resveratrol content in CNP extract.

DPPH ^1^	ABTS ^1^	Total Phenolic Content ^2^	Total Flavonoid Content ^3^	Resveratrol Content ^4^
37.65 ± 4.72	56.82 ± 0.86	383.07 ± 1.83	43.71 ± 1.47	1.51 ± 0.07

Values are means ± SD of the three independent experiments. ^1^ % Scavenging activity; ^2^ mg gallic acid equivalent/g extract; ^3^ mg quercetin equivalent/g extract; ^4^ mg/100 g DW.

## Data Availability

The data are contained within the article. The mRNA sequence data used in this study have been deposited in the DNA Data Bank of Japan (DDBJ) database under accession numbers (accessed date: 19 April 2022) and active repository links as follows: SOD1: LC706445 [http://getentry.ddbj.nig.ac.jp/getentry/na/LC706445]; SOD2: LC706446 [https://getentry.ddbj.nig.ac.jp/getentry/na/LC706446]; GPx1: LC706447 [https://getentry.ddbj.nig.ac.jp/getentry/na/LC706447]; CAT: LC706448 [https://getentry.ddbj.nig.ac.jp/getentry/na/LC706448].

## Sample Availability

Samples of the compounds are not available from the authors.
